# The Insect Eye: From Foundational Biology to Modern Applications in Pest Management

**DOI:** 10.3390/insects17020167

**Published:** 2026-02-02

**Authors:** Marianna Varone, Paola Di Lillo, Francesca Lucibelli, Gennaro Volpe, Angela Carfora, Sarah Maria Mazzucchiello, Serena Aceto, Giuseppe Saccone, Marco Salvemini

**Affiliations:** Department of Biology, University of Study of Naples Federico II, 80100 Naples, Italy; paola.dilillo@unina.it (P.D.L.); francesca.lucibelli@unina.it (F.L.); gennaro.volpe2@unina.it (G.V.); angela.carfora@unina.it (A.C.); sarahmaria.mazzucchiello@unina.it (S.M.M.); serena.aceto@unina.it (S.A.); giuseppe.saccone@unina.it (G.S.); marco.salvemini@unina.it (M.S.)

**Keywords:** insect vision, phototransduction, phototaxis, pest management

## Abstract

The ability of an animal to perceive its visual environment underpins many behaviors essential to survival, including navigation, foraging, predator avoidance, and recognition of conspecific individuals, making vision a critical element of both reproductive success and survival itself. In insects, eyes have evolved widely, shaped by different habitats and lifestyles, with striking examples such as the high-resolution diurnal vision of dragonflies, which enables rapid detection of prey and environmental features, in contrast with the highly sensitive nocturnal optical system of hawkmoths, which specializes in capturing even single photons. At the core of this diversity is a fundamental trade-off: at one extreme lies sensitivity, the ability to perceive visual stimuli, even under poor lighting conditions. At the other extreme, acuity, is the ability to resolve fine spatial details. This review seeks to synthesize current knowledge of insect visual systems, from their evolutionary origins to the developmental processes so far identified, from cellular organization to their role in behavior, to provide insights for designing novel, targeted, and sustainable vision-based technologies for the control of pest insects.

## Simple Summary

This review summarizes current knowledge of insect visual systems and how these visual abilities have become pivotal to everyday insect behaviors, such as flying, food or partner seeking, and predator avoidance. The diversity of the structures of the insect eye faces a fundamental trade-off: eyes must either be capable of high spatial acuity or highly sensitive to low light at night. We bring together knowledge from studies spanning the basic structure and genetics of the insect eye to brain image processing, highlighting the potential of this information for the design of new, smart, and sustainable methods of harmful insect control, such as highly selective light traps and visual deterrents.

## 1. Comparative Anatomy of Insect Visual Organs

The visual system of insects is a very important field of study in biology, providing profound insights into the astonishing adaptability and evolutionary innovation of life. Crucially, the extensive differences in the structure and function of these eyes are studied precisely because vision is the primary sensory modality governing nearly all essential behaviors, including foraging, navigation, predator avoidance, and successful reproduction [1].

**Figure 1:**
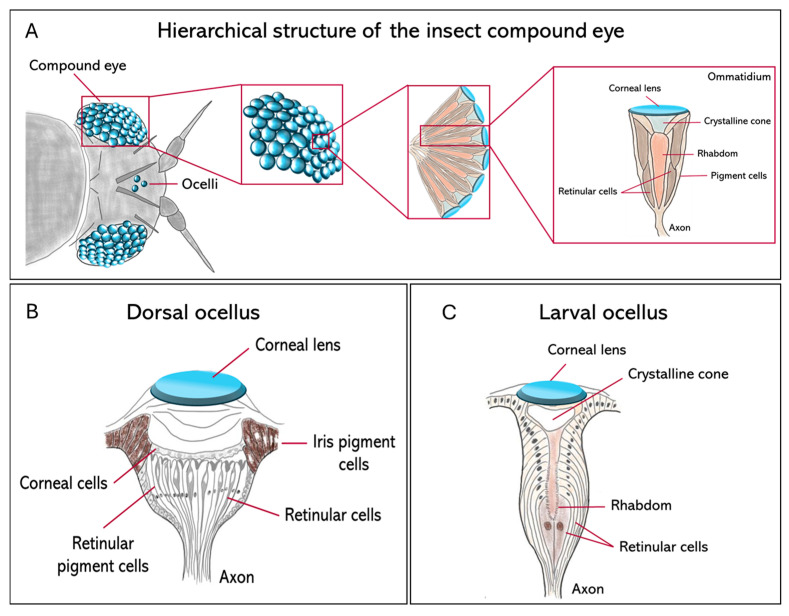
Comparative anatomy of the principal insect visual organs. Schematic diagrams show the structural organization of the compound eye, dorsal ocellus, and larval stemma in insects. (**A**) The hierarchical structure of the apposition compound eye. The arrangement of individual photoreceptive units (ommatidia) that forms the whole organ is represented. An ommatidium is made of a corneal lens and a crystalline cone, which focus light onto the rhabdom, which is formed by the microvilli of several retinular cells; (**B**) the adult dorsal ocellus, characterized by a single large corneal lens overlying a broad, continuous retina of retinular cells; (**C**) the larval stemma, a simple photoreceptive organ in holometabolous larvae, composed of a lens and a small number of retinular cells.

Insect visual systems differ widely in both structure and function, with over half a billion years of evolutionary change. They include three main types of eyes, compound eyes (Figure 1A), ocelli (Figure 1B), and larval stemmata (Figure 1C), each adapted to different ecological and developmental contexts.

Compound eyes serve as primary vision tools in mature insects and are constituted by repeating units called ommatidia (Figure 1A). Their number and morphology vary significantly between species. For example, parasitoid wasps such as *Trichogramma evanescens* and *Megaphragma mymaripenne* (Hymenoptera, Trichogrammatidae) may have only a few dozen ommatidia [2,3], while dragonflies such as *Anax junius* (Odonata, Aeshnidae) reach around 30,000 per eye, providing them with exceptionally high spatial resolution and strong motion sensitivity [4,5]. In the case of blowflies like *Calliphora vicina* (Diptera, Calliphoridae), there are large, specialized frontal ommatidia to facilitate high-acuity tracking in aerial predation [6]. Three major compound eye designs have evolved across insects as solutions to solve the classical trade-off between sensitivity and acuity. Apposition eyes are typical for diurnal insects, such as honeybees, *Apis mellifera* (Hymenoptera, Apidae), and prioritize high acuity by optically isolating individual ommatidia; superposition eyes, characteristic of nocturnal moths, including *Deilephila Elpenor* (Lepidoptera, Sphingidae), maximize sensitivity at the expense of acuity by allowing multiple lenses to focus light onto shared photoreceptors; finally, neural superposition eyes, found in dipterans such as *Musca domestica* (Diptera, Muscidae), represent a powerful compromise: their sensitivity (the ability to detect light under low-light conditions) increases without a significant decline in acuity (the ability to distinguish fine spatial details) [6,7]. Despite functional variability, compound eyes commonly exhibit a conserved basic architecture: each ommatidium contains four cone cells and eight photoreceptors. Recently, the use of synchrotron X-ray microtomography has enabled the non-invasive 3D reconstruction, at very high resolution, of such internal structures in a variety of groups, including Coleoptera [8] and Lepidoptera [9], with implications for understanding their optical anatomies.

In addition to compound eyes, most insect species exhibit a pair, or a triad, of simple dorsal eyes called ocelli, which are ancient visual organs specialized for flight control rather than high-resolution image formation (Figure 1B). These light-sensitive organs are found in flies, grasshoppers, and mantids, among others, and are usually arranged in a three-lens configuration. They contain defocused optics and are very sensitive to extensive changes in illumination from the entire sky or horizon [10,11]. The primary function of the ocelli is to stabilize flight through rapid visual feedback, thereby maintaining the insect’s midair orientation. Neural recordings from ocellar pathways usually show a phasic response, meaning that they react to changes in light intensity (the amount of light emitted or reflected by a light source) rather than to the overall brightness (the subjective perception of light level as experienced by the visual system). This makes the ocelli well adapted to estimating the angular speed of the visible scene, with a significantly shorter latency than is feasible with compound eyes [12]. Compared to the slower image-forming input from compound eyes, the rate-dependent signal from the ocelli provides a far more rapid route to flight stabilization. This quick input is especially important during sudden orientation shifts, when the insect can carry out continuous high-speed corrections to stay upright and oppose rotational disturbances during flight [12,13]. This is particularly important for insects such as the honeybee, *A. mellifera*, which lacks the gyroscopic halteres that dipteran flies use for stability [13]. Even though the flight of honeybees is still possible in cases when their ocelli are occluded, the precision of flight and the ability to maintain a course stably are significantly reduced, thus underlining the role of ocelli in providing an immediate estimate of angular velocity necessary for robust flight control.

Larval visual organs, called stemmata, represent another example of evolutionary flexibility in insect vision (Figure 1C). Stemmata are simpler in structure than compound eyes but often retain homologous cell types, such as cone cells, pigment cells, and retinula cells [14], while retaining a high degree of evolutionary plasticity [15]. In *Drosophila melanogaster* (Diptera, Drosophilidae), the Bolwig organ is a rudimentary larval visual system that shares developmental gene expression with adult photoreceptors [11]. In *Tribolium castaneum* (Coleoptera, Tenebrionidae), the transcription factor *glass* is expressed in both the stemmatal and ommatidial photoreceptors, supporting their shared origin [16]. In the most highly miniaturized forms, such as first-instar larvae of *Strepsiptera*, ultrastructural reconstructions show functional but highly reduced eye structures [17].

As extensively reviewed by Gilbert [18], over evolutionary time, the stemmata have diversified in two principal directions: fusion and expansion. For instance, the stemmata of the sawfly, *Perga affinis* (Hymenoptera, Pergidae), represent a fusion of multiple ommatidia into a single-lens system that can accommodate hundreds of photoreceptors [19]. In contrast, in tiger beetle larvae, *Cicindela chinensis* (Coleoptera, Cicindelidae), the stemmata evolved through the dramatic enlargement of individual ommatidia, creating tiered arrays of thousands of photoreceptors behind a single, massive lens [20]. This kind of layered retina enables such larvae to scan their environments, thus creating a high-resolution image of their surroundings to find prey—a strategy fundamental to stemmata functional versatility [18]. This is also the case for swallowtail butterfly larvae, *Papilio xuthus* (Lepidoptera, Papilionidae), which exhibits a similar tiered arrangement of photoreceptors that cooperate in the formation of a basic image [20,21]. Interestingly, these optical modifications, especially the use of low F-numbers (which represent the proportion of the focal length with respect to the aperture diameter and, therefore, signify a wide opening capable of maximizing light collection), seem to be an adaptation for delivering maximum visual performance at sites of scant visibility, where photon supply is minimal [14].

The complexity of vision in larvae is highest in aquatic environments. In the diving beetle, *Thermonectus marmoratus* (Coleoptera, Dytiscidae), for example, bifocal lenses within the stemmata project onto two distinct retinas, enabling parallel processing of spatial and spectral information, including polarized light [22]. Indeed, recent fossil finds of Cretaceous lacewings are consistent with the proposition that refined binocular vision and the convergent evolution of complex simple eyes occurred very early on in the history of holometabolous insects [15]. A further increase in structural complexity is encountered in the larval visual system of *Aedes aegypti* (Diptera, Culicidae), which has five discrete stemmata on either side: dorsal anterior, dorsal posterior, central, ventral, and satellite. Each has fused rhabdoms formed by converging photoreceptors that differentially express two long-wavelength-sensitive rhodopsins: Aaop3, expressed in all five, and Aaop7, confined to the central and satellite units [23].

These examples collectively demonstrate the impressive evolutionary adaptability of basic visual components, from the high-resolution compound eyes of *A. junius* and *C. vicina* to the specialized larval stemmata of *T. marmoratus* and *A. aegypti*. These structural variations did not occur randomly but were shaped by ecological pressures and distinct developmental pathways. Overall, they stress the impressive adaptive potentials of insect eyes across life stages, habitats, and evolutionary lineages.

## 2. Evolutionary and Morphological Diversity of Insect Eyes

The evolution of the insect visual system is highly diverse and is influenced by evolutionary history, ecological context, and life stage. Although certain model groups of organisms, such as *Drosophila* and *Apis*, have contributed significantly towards understanding vision, it is important to appreciate that these organisms are only a limited representation of the insect world.

### 2.1. Evolutionary Origins and Optical Strategies

The compound eye is representative of the arthropod body plan and had its origins some 500 million years ago, during the Cambrian explosion [24,25]. Enough evidence from early fossil arthropods (such as trilobites and radiodonts) has already suggested that the ancestral visual organ was a modular structure that consisted of repeating optical units. This ancient design has largely diversified in different lineages over the course of evolution to satisfy ecological niches [26].

Although the basic underlying modular architecture is conserved, important variations have emerged in the optical mechanisms―e.g., apposition vs. superposition. In the ancestral anatomical apposition eye, as in the diurnal insect, optical isolation of each ommatidium by pigment cells ensures high resolution. Conversely, the superposition eye is an important innovation for nocturnal lineages: here, by moving the screening pigments away from the tip, many lenses may focus their light on a single rhabdom, strongly increasing photon capture at the expense of resolution [24].

Comparative studies across arthropods reveal deeper evolutionary patterns in eye development. In particular, in all euarthropods, the mode of retinal growth diverges, although they have a proliferation zone at the edge of the developing eye field. In Trilobita (extinct marine arthropods), Xiphosura (marine chelicerates), and Diplopoda (terrestrial myriapods), eye formation occurs in a “row-by-row” fashion, with large ommatidia added sequentially and existing units continuing to enlarge after formation through intercalary growth [27,28,29]. In contrast, members of Tetraconata (Hexapoda + Crustacea) develop their eyes according to a “morphogenetic front” model. In this model, new ommatidia are generated from a mitotic zone and organized by a morphogenetic furrow that moves across the eye field, spatially and temporally separating proliferation from differentiation. This process has been thoroughly characterized in *Drosophila* and occurs in crustaceans such as *Homarus americanus* (Decapoda, Nephropidae) and *Procambarus clarkii* (Decapoda, Cambaridae), although the formation of pre-clusters is often less well defined in these species [29,30,31,32,33].

**Figure 2:**
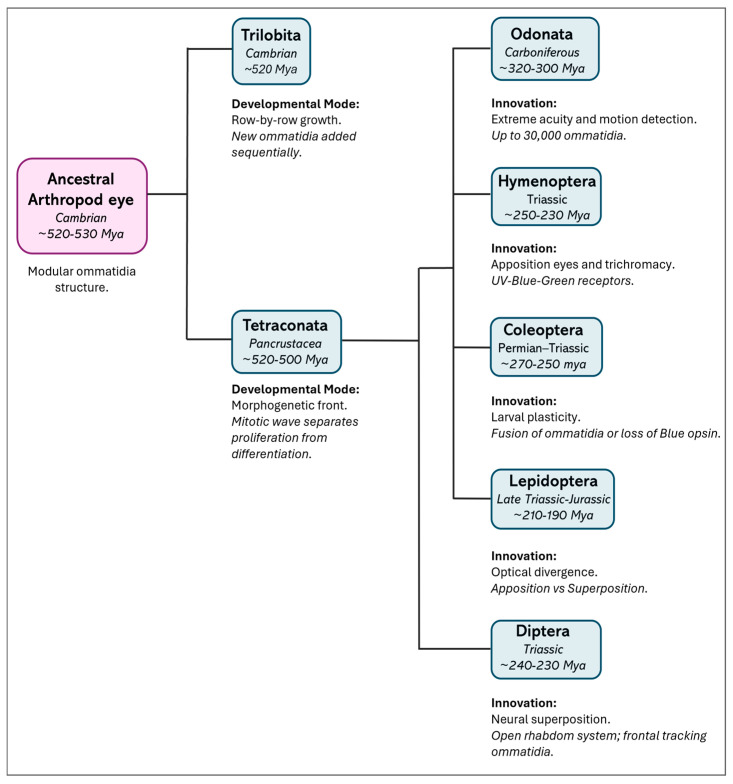
Evolutionary divergence and key innovations in the insect visual system. The schematic illustrates eye diversity from the common arthropod ancestor to existing insect orders. Divergence times (in italics) are approximate estimates based on phylogenomic data [35]. A major developmental split occurred between the extinct group Trilobita, in which eyes developed by adding new rows (“row-by-row” development) [27], and Tetraconata [29,34] (Crustacea + Hexapoda), where a layer of dividing cells (the “morphogenetic front”, driven by a mitotic wave) allowed for the development of a visual system called “neural superposition”—where signals from several neighboring units are combined to improve sensitivity. Hexapods further adapted their vision for different environments: “Apposition eyes,” found in Odonata (dragonflies and damselflies) and Hymenoptera (bees, wasps, ants), have separate units for each focus point, offering high resolution and color vision [4,36]. Coleoptera (beetles) [18,19] and Lepidoptera (butterflies and moths) [21] use simple eyes (strong stemmata) as larvae and, as adults, adjust vision for various light levels—moths develop “superposition eyes,” where multiple units gather light to see better in the dark. Diptera (flies) [37] use “neural superposition” to boost sensitivity while maintaining sharpness.

Interestingly, the centipede *Scutigera* (order Scutigeromorpha) shows a middle-type development, where its proliferation zone forms discrete proto-ommatidial clusters without a continuous morphogenetic front, suggesting a transitional developmental strategy between ancestral and derived states [29,34]. These developmental trajectories broadly parallel phylogenetic relationships and support the monophyly of Tetraconata, characterized by a fixed ommatidial blueprint and a spatially structured developmental process (Figure 2).

In particular, the visual systems of insects are very diversified due to ecological demands and lifestyles. This diversity is strongly supported by the evolution and functional differentiation of the opsin genes. The process of gene duplication enabled insects to extend their spectral capabilities from a UV/blue–green-sensitive ancestral receptor array to one that can tolerate red light or even specialized UV receptors for navigation [38]. Thus, the honeybee, *A. mellifera*, develops large, forward-facing compound eyes that harbor three types of photoreceptors sensitive to UV, blue, and green wavelengths. This is a trichromatic configuration that allows for accurate color vision and acceptable spectral discrimination, important for foraging in general and flower identification in particular [39]. At the other extreme, the red flour beetle, *T. castaneum*, adapted to living in dark and concealed habitats, has undergone significant visual reduction. Its compound eyes lack blue-sensitive photoreceptors because they lack the corresponding opsin gene and its processing machinery, resulting in a dichromatic system based on UV- and long-wavelength-sensitive opsins. Uniquely, *Tribolium* co-expresses two long-wavelength opsins across the retina; this may be a strategy to improve achromatic contrast sensitivity instead of color discrimination and is a likely adaptation to low-light conditions [40]. A contrast between the red flour beetle and the honeybee shows how distinct environmental demands shape vision, despite their similar genetic origins. During the day, the honeybee relies on precise navigation for pollination, forming compound eyes through placode-type tissue development alongside a regular pattern of ommatidia maturation [41]. In sharp contrast, *T. castaneum*, a cryptozoic pest beetle that thrives in the perpetual darkness of stored grain, exhibits altered developmental dynamics, consistent with its reduced visual ecology. Hence, both insects have lifestyles that have taken their visual capabilities in fundamentally opposite directions. Table 1 provides a comprehensive representation of this vision in key insects.

### 2.2. Developmental Mechanisms: From Embryos to Adults

Although the output differs, the basic mechanism of eye development depends on a conserved genetic toolkit. In holometabolous insects such as *Drosophila*, compound eyes form post-embryonically from a specialized epithelial primordium, the eye–antennal imaginal disc. During larval development, a wave of differentiation (the morphogenetic furrow) moves across this epithelial primordium, organizing the recruitment, specification, and patterning of cells into the exact crystalline arrays of ommatidia [60].

The key regulatory gene at the top of the developmental hierarchy is *Pax6* (the *eyeless* gene in *Drosophila*), which is conserved throughout the animal kingdom and is essential for forming the early eye structure. Below *Pax6*, a complex gene regulatory network (GRN) controls every subsequent step in development, from cell proliferation ahead of the furrow to terminal differentiation of photoreceptor neurons, and the expression of specific rhodopsin genes that define their spectral sensitivities, whose regulatory elements have become essential tools for transgenesis and gene function studies in insects [61]. Because these GRNs coordinate not only structural assembly but also evolutionary adaptation, even minor mutations can trigger significant changes in vision. However, these biological programs can be associated with different anatomical contexts. For example, while in *Drosophila*, eye organs develop from the inner imaginal discs; in other organisms such as *T. castaneum* or *A. mellifera*, eye organs develop from lateral placode-like epithelial tissues. Nevertheless, these biological programs entail similar differentiation programs, in that they both spread from the posterior to anterior in both contexts [6].

It should be noted that the conserved nature of this developmental toolkit does not imply rigidity. In fact, small evolutionary changes in the timing, spatial distribution, or level of gene expression within the GRN can generate the extensive morphological diversity observed among insect eyes. Such modifications may underline major structural and functional innovations, including differences between apposition and superposition optics [37], or the emergence of localized high-acuity regions such as foveas, small central retinal areas with the highest density of photoreceptors that allow for precise and detailed vision. In that respect, variation in developmental programs is directly linked to the evolution of eye designs suited to ecological roles.

The transformation from the larval to the adult visual system during metamorphosis is a significant developmental event in which the simple larval eyes (stemmata) are replaced by the much more complex compound eyes and ocelli of the adult, reflecting the shift to a very different way of life and visual ecology [62]. This developmental change is regulated by a highly sensitive hierarchy of genetic and molecular cues.

Reduction is most salient in the larval stage of holometabolous insects. While compound eyes usually appear in adults, larvae develop simplified organs called stemmata. In more basal holometabolous taxa, such as the Mecoptera order, stemmata still exhibit a modular organization like that of compound eyes. In more derived ones, such as *Diptera*, stemmata are further reduced to single structures, as in the case of the *Bolwig* organ in *Drosophila*, which stays photoreceptive despite its extreme morphological reduction [11,63].

The evolutionary development of stemmata illustrates how a conserved developmental toolkit can give rise to a diverse range of eye morphologies. Stemmata arise from embryonic visual primordia, homologous with those that produce compound eyes, and recruit similar cell types, including photoreceptors, cone cells, and pigment cells. In different species, these may form enlarged single-lens units or more complex, fused image-forming structures. For example, larvae of *T. marmoratus* develop complex camera-type eyes with tiered retinas and bifocal lenses, all derived from typical ommatidial cell populations. This case helps in illustrating how modifications in developmental timing and patterning can give rise to novel visual architectures suited to ecological pressures [14].

In addition to the elaboration of complex compound eyes, reduction and remodeling are standard features of insect visual development. Many such changes are ecological in nature, representing shifts to parasitic, subterranean, or larval lifestyles, in which full visual capacity no longer confers a selective advantage. One of the most dramatic examples occurs in the *Strepsiptera* order, in which the ancestral compound eye has been remodeled into independent image-forming “eyelets,” each acting as an individual single-lens unit, an apparent case of evolutionary reuse [52].

Taken together, these insect visual systems show how highly conserved genetic pathways can change multiple times in response to different environmental demands. In one lineage, these pathways yield complex, trichromatic eyes suited for flower recognition and navigation; in another, they underlie dramatic visual reductions and radical reorganization. Rather than static designs, insect eyes represent dynamic products of evolutionary tinkering, shaped and reshaped by lifestyle, environment, and selection.

## 3. Molecular and Cellular Basis of Vision

**Figure 3:**
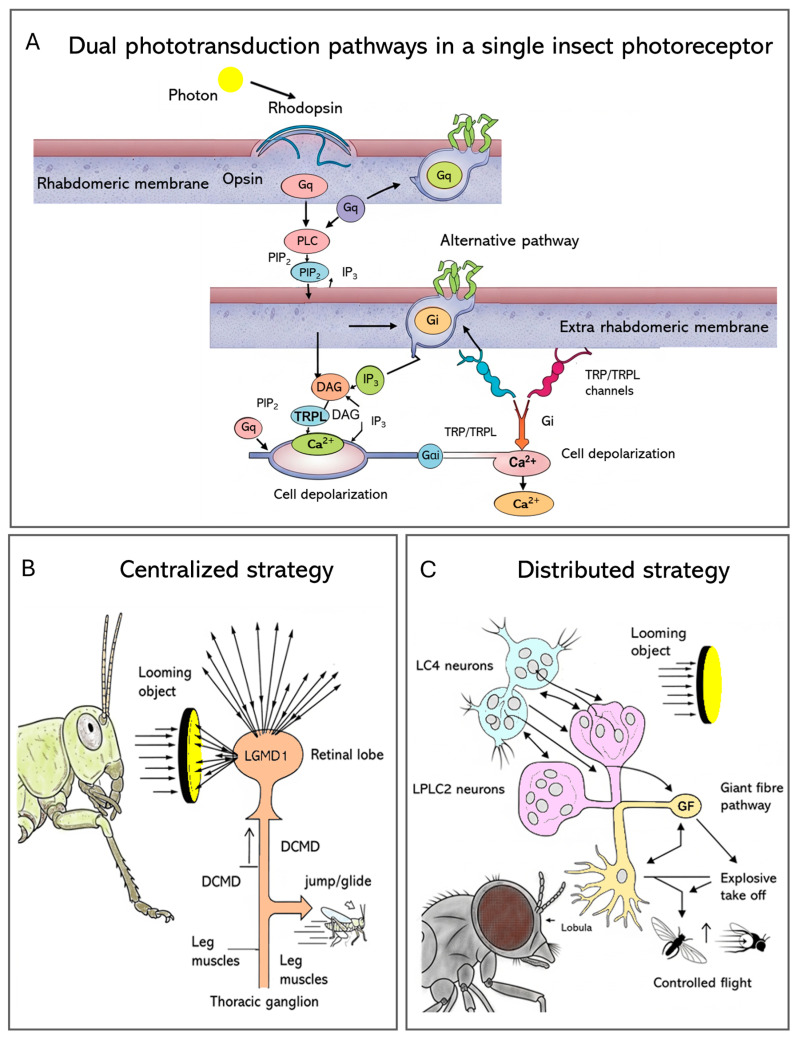
The molecular and neural basis of insect vision. (**A**) The canonical Gq/PLC or the alternative Gi/Opn3 phototransduction cascade is able to convert, within a photoreceptor, light into an electrical signal. (**B**) Locusts use a single Lobula Giant Movement Detector (LGMD1) neuron that gathers broad visual data and then activates the DCMD to drive fast escape reactions. (**C**) In *Drosophila*, two selective neuron groups (LC4 for angular velocity, LPLC2 for angular size) relay information to the Giant Fibre (GF) pathway, where combined input shapes appropriate getaway decisions.

Insect eyes begin with specialized light-sensitive cells (PRCs) in the retina, where phototransduction converts light into an electrical signal (Figure 3A). Light detection occurs within tiny structures called rhabdomeres: microvillar compartments filled with proteins known as opsins, which are linked to retinal molecules. When a photon hits, the retinal shifts shape, causing the attached opsin (typically a rhodopsin) to change form and switch on a Gq-type G heterotrimeric protein complex [64]. That activation leads to phospholipase C (PLC) being activated, breaking down PIP_2_ into two components: Diacylglycerol (DAG), along with Inositol 1,4,5-trisphosphate (IP_3_). Second, messengers switch on TRP and TRPL ion channels, causing calcium entry plus membrane depolarization (Figure 3A). Because the q/PLC pathway acts quickly, it enables rapid vision processing, essential for actions such as flying [6,65].

Signal transmission, however, also requires a quick turn-off response for the light signal, which sets the temporal resolving power of vision [66]. After photon absorption, activated rhodopsin, also known as metarhodopsin, needs rapid deactivation to avoid chronic signaling [67]. The visual pigment requires rejuvenation via visual cycle renewal, plus light adaptation via screening pigment granule transport to rhabdomeres [68].

Variations in how insects detect light, colors, or movement—differing between species or within them—are influenced by changes in the evolutionary diversification and expression patterns of rhodopsin genes, helping in the understanding of sensory differences across insect groups [36].

Indeed, color vision comes from different opsins being active in specific PRCs, usually tuned to UV, blue, green, or longer wavelengths. For instance, the *D. melanogaster* eye, often used to study rhabdomeric light sensing, organizes its eight photoreceptors (R1–R8) into repeating units called ommatidia. Instead of sharing pigments, R1–R6 use Rh1 (~480 nm peak), helping detect movement. In contrast, central R7 and R8 carry separate opsin types that tell colors apart, especially differentiating between ultraviolet and green light (~330–520 nm) [69,70]. Even though these roles differ, every cell uses a nearly identical signaling path when catching light. Also, their response strength per photon stays consistent, so they work well over a broad range of light intensities [71]. The positioning and activity of these opsins adjust precisely to different insect environments, depending on species-specific needs [6].

The visual system of the bumblebee, *Bombus impatiens* (Hymenoptera, Apidae), illustrates the hierarchical processing of visual information, from photoreceptor signals relayed from the lamina through the medulla and lobula to the lateral protocerebrum [72]. The segregation is both functional and anatomical: distal lobula neurons detect movement and send outputs backward, while proximal lobula neurons process color. This organization results in a pattern where anterior protocerebral neurons are tuned to color, and posterior neurons are tuned to motion [72]. Recent findings reveal that phototransduction is more complex than previously believed. In *A. aegypti*, an opsin homologous to vertebrate *Opn3* (called *AeOpn3*) was found in R7 cells, but it was localized not to the rhabdomere, as expected, but to the extra-rhabdomeric cytoplasm. Unlike classical visual opsins, *AeOpn3* activates a Gi-type G protein and not the canonical Gq. That means that one photoreceptor may run two separate light detection systems at once: a fast Gq/PLC pathway for rapid, image-forming vision, while a slower Gi/Opn3 pathway handles tasks such as daily rhythm adjustment or prolonged light exposure [73]. These dual phototransduction mechanisms indicate a functional division within the same cell and enable parallel processing of visual information, with modulatory responses. Molecular and cellular signaling in photoreceptors provides the critical input to higher-order neural circuits that integrate visual information and drive rapid behavioral responses. There is further evidence for spatial and functional specialization in *Anopheles stephensi*, where *MosOpn3* (a homolog of *Opn3*) is co-expressed with the short-wavelength-sensitive opsin *Asop9* in dorsal and ventral R7 cells. This may suggest that two different G-protein-coupled pathways, Gq and Gi/Go, act in parallel within the same PRC and enhance spectral discrimination or modulate photoreceptor sensitivity depending on ambient light conditions [73].

Comparative studies of fireflies like *Ellychnia corrusca* and *Lucidota atra* (Coleoptera, Lampyridae) highlight how flexibly their light-sensing systems can evolve. While transcriptomic analyses still show limited opsin diversity, with UV- and long-wavelength-sensitive opsins dominating several downstream components of the cascade that have undergone adaptive diversification, genes such as *inaD*, *trpl*, *arr2*, *Gq*, and *ninaC* are under positive selection, especially in diurnal lineages such as *Ellychnia* and *Lucidota*. The scaffold protein *inaD*, central to assembling the rhabdomeric signaling complex, has duplicated in multiple species, possibly supporting region-specific expression or novel functions. These adaptations likely reflect selection for improved visual performance under high-light conditions [74].

The molecular-level tuning of the phototransduction apparatus in nocturnal species provides the direct physiological basis for remarkable nocturnal behaviors discussed later, such as hawkmoth color discrimination under starlight and the celestial navigation of single-photon-sensitive dung beetles. In Lepidoptera, the canonical phototransduction cascade is conserved but features lineage-specific modifications. The butterflies and moths share the Gq/PLC–TRP/TRPL cascade but also express unique opsins, such as *UnRh* and RGR-like opsins. *UnRh*, expressed in cone and pigment cells of *Heliconius melpomene* (Lepidoptera, Nymphalidae), shows a phylogenetic relation to squid retinochromes and may participate in the photoisomerization of retinal during rhodopsin regeneration. Furthermore, gene expression studies show that *trp* and *trpl* channels are upregulated in diurnal butterflies, consistent with high-light vision. In contrast, low *trp* expression in nocturnal moths, such as *Manduca sexta* (Lepidoptera, Sphingidae), suggests adaptation to dim light. Expression of other genes, including *Calx* and *Nckx30C* (involved in Ca^2+^ extrusion), further adjusts photoreceptor physiology to environmental light levels [65].

One of the most important tasks of any visual system is quickly detecting approaching objects to trigger escape, and insects have developed highly specialized neural circuits for this purpose. Comparing locusts and flies shows two different but equally effective evolutionary strategies for solving this problem.

In the locust, this function is mediated by the Lobula Giant Movement Detector (LGMD) (Figure 3B). A single, large interneuron, LGMD1, integrates visual information across the entire eye, making it extremely sensitive to the coherent expansion of a looming object. Its robust, reliable spiking response directly drives the Descending Contralateral Movement Detector (DCMD) neuron, which acts as a “command neuron” to trigger stereotyped escape behaviors like jumping or gliding [75]. This system employs a highly centralized architecture, with just a single neuron in charge of detecting threats and initiating escape. In contrast, *Drosophila* employs a more distributed, parallel approach for the same task (Figure 3C), with looming detection split between two groups of smaller neurons: Lobula Columnar (LC4—neurons that respond to the angular velocity of the moving object) and Lobula–Plate Lobula Columnar (LPLC2—neurons that encode its angular size). Their outputs are combined by the Giant Fiber (GF) escape pathway, resulting in computation that allows the fly to choose an appropriate escape response: launching into a quick, explosive takeoff when danger is close or beginning a more relaxed, deliberate flight when the threat seems less immediate [75].

This comparison between locusts and flies showcases how two different neural architectures, one centralized and the other distributed, come together to tackle the same basic survival challenge, highlighting functional specialization in the insect brain.

## 4. Physiological Mechanisms of Visual Processing

Insects have sophisticated visual systems that assist them in various activities—such as finding food, avoiding predators, moving, hunting, and maintaining daily rhythms. These functions are driven by specific brain pathways and tiny-level changes that interpret movement, color, distance, or light direction.

### 4.1. Adaptations for Sensitivity and Acuity

Visual features balance clarity with low-light sensitivity: diurnal insects enhance detail perception with closely packed eye units, quick signal conversion, and small viewing zones; nocturnal insects prefer detecting faint light through layered lenses, delayed cell responses, and signal integration in nerves. For example, elephant hawkmoths detect movement under starlight by combining spatial and temporal signals in a region of their optic lobe, allowing them to perceive motion even in star glow [76]. Although seeing color is not limited to daylight, hawkmoths and carpenter bees can discriminate colors in dim conditions thanks to three-receptor sight and amplified receptor output [77,78].

Some insects, like the night-active bee, *Megalopta genalis* (Hymenoptera, Halictidae) or the ant, *Myrmecia pilosula* (Hymenoptera, Formicidae), which initially have simple eye types, developed larger lenses and broader rhabdoms over time; this adaptation helps gather more light, showing how different species arrive at similar solutions for seeing in darkness. In terms of function, nocturnal insects’ light-detecting cells are built for maximum responsiveness and consistency. Instead of relying on speed, their receptors amplify signals more strongly—one photon produces a larger voltage spike, called a quantum bump, compared to day-living relatives. Moreover, these spikes last longer, which makes overlapping them easier and improves signal clarity during low-light conditions [79].

### 4.2. Color and Polarization Circuitry

Although sensitivity to light is necessary for survival when light levels are low, wavelength detection facilitates complex light interactions during the daytime. Color vision also varies across taxa; for example, fruit flies process color contrasts early on in a brain region called the medulla [80], and butterflies like *P. xuthus* detect four colors and use opposing signals in their first visual layer, the lamina [81]. Some species, like *Graphium sarpedon* (Lepidoptera, Papilionidae), possess up to 15 distinct photoreceptor types for fine spectral resolution [82].

These abilities are enhanced by physical modifications in their eyes—such as colored filters and uneven eye unit patterns—which improve color judgment during feeding or egg-laying.

In addition to chromatic cues, many insects exploit a visual modality invisible to humans: polarized light. Polarization vision functions through special cells at the top edge of the eye, along with separate pathways in the brain’s medulla: the dorsal rim area (DRA). The DRA is a unique part of its compound eye, showing how body features can enable precise navigation. Within the DRA, light-detecting cells are arranged in a fan-like pattern, with tiny structures oriented at right angles; this configuration detects the direction of polarized light with high sensitivity. Polarization vision is processed by these specialized DRA ommatidia and separate medulla circuits, creating a distinct pathway from other visual inputs.

### 4.3. Motion Detection and Neuromodulation

Among visual modalities, motion detection is among the most thoroughly characterized. Foundational models by Hassenstein and Reichardt (1956) [83] inform our understanding of optic flow processing in *D. melanogaster*, where Lobula Plate Tangential Cells (LPTCs) encode direction-selective signals for flight stabilization [84,85].

In hoverflies, *Eristalis tenax* (Diptera, Syrphidae), descending neurons that spot tiny moving objects work separately from those sensing broad visual motion—this split helps manage flight [86]. Instead of relying on movement clues, mantises see depth using both eyes together, thanks to special nerve cells in their optic lobe that judge how far away prey is by image differences [87].

On a more detailed scale, recent studies have revealed that muscles moving the retina to stabilize gaze in *Drosophila* [88] and a conserved system of photoreceptor microsaccades that enable hyperacute 3D vision beyond the limits predicted by static morphology [88].

Moreover, processing and interpretation of visual cues are under dynamic neuromodulatory control. Neuromodulators like octopamine and serotonin further reconfigure the sensitivity of the visual system to align visual processing with the animal’s internal state and behavioral context [89]. This is also triggered by locomotion, as evidenced, for instance, by increased visual sensitivity during flight in *Drosophila* [88]. At the molecular level, the expression of specific ion channels—such as TRP, TRPL, voltage-gated sodium channels, and fast potassium channels—ensures the rapid signal transduction and propagation required for behaviorally relevant visual tasks [90,91].

## 5. Visually Guided Behaviors in Ecological Contexts

Flying insects heavily depend on visual cues for navigation, avoiding obstacles, foraging, and homing. The visual information obtained in flight is further processed by highly specialized circuits that underpin basic behaviors such as phototaxis and optomotor responses, as well as higher-order cognitive skills, including spatial memory, landmark recognition, and path integration.

### 5.1. Navigation and Homing

Celestial signals are crucial for insects to find direction. The desert ant, *Cataglyphis fortis* (Hymenoptera, Formicidae), is able to detect polarized light from the sky using the DRA, showing how body features can enable precise navigation. Similarly, desert ants such as *Veromessor pergandei* (Hymenoptera, Formicidae) respond to sky polarization not only while searching alone but also when moving along chemical paths, showing that these sky-related systems work even in socially guided contexts [92]. This strategy is not unique to ants: nocturnal and diurnal dung beetles, such as *Scarabaeus lamarcki* (Coleoptera, Scarabaeidae), use polarized light along with sky brightness changes to move straight, changing course by tracking sunlight, moonlight, or how light scatters across the sky, depending on environmental conditions [93,94]. Some species can even use the Milky Way as a navigational reference [95]. These behaviors highlight the adaptability of polarization vision in diverse ecological scenarios, especially when integrated with social cues [92].

These navigational systems operate within a hierarchical framework: path integration provides rough position, whereas scene recognition helps pinpoint exact spots [96]. Many insect species practice path integration, combining celestial compass cues with an odometer based on optic flow or stride count, to continuously update the home vector [97,98].

### 5.2. Flight Control and Predation

In addition to compass navigation, vision is essential for immediate motor control. A common denominator for many taxa is the analysis of the pattern of visual motion produced by self-motion, also known as optic flow, which conveys essential information for flight control, landing, and odometry [99].

During flight, they compare motion across both eyes, which helps them stay centered when moving through tight spaces. Meanwhile, how fast images grow on their retina controls stopping behavior before obstacles or during landings [100,101,102]. To improve perception, insects use active vision strategies, such as saccadic flight patterns that distinguish rotational from translational optic flow, allowing for more accurate distance estimation during intersaccadic intervals [103].

This core relationship between structure and function is well illustrated in the divergent seeking strategy of predatory insects. Dragonflies, for instance, utilize their high-acuity dorsal foveas to track and intercept prey with extreme precision [104]. In cluttered environments, the bumblebee, *Bombus terrestris* (Hymenoptera, Apidae), deploys computational flexibility by switching from an averaging strategy to a maximum pooling of optic flow, thus responding to the most imminent threats [105].

Finally, visual cues play a pivotal role in social interactions, such as mate preference in *Heliconius melpomene* and *H. timareta* (Lepidoptera, Nymphalidae), where visual attraction can be modulated by genetic and social factors [106,107,108].

## 6. The Application of Light-Based Technologies in Pest Management

The principles of insect visual ecology currently offer a robust and sustainable pathway for the management of pests and vectors. Leveraging innate responses to light has enabled the development of a range of effective control strategies, evolving from broad approaches to specific, technologically advanced solutions [109].

**Figure 4:**
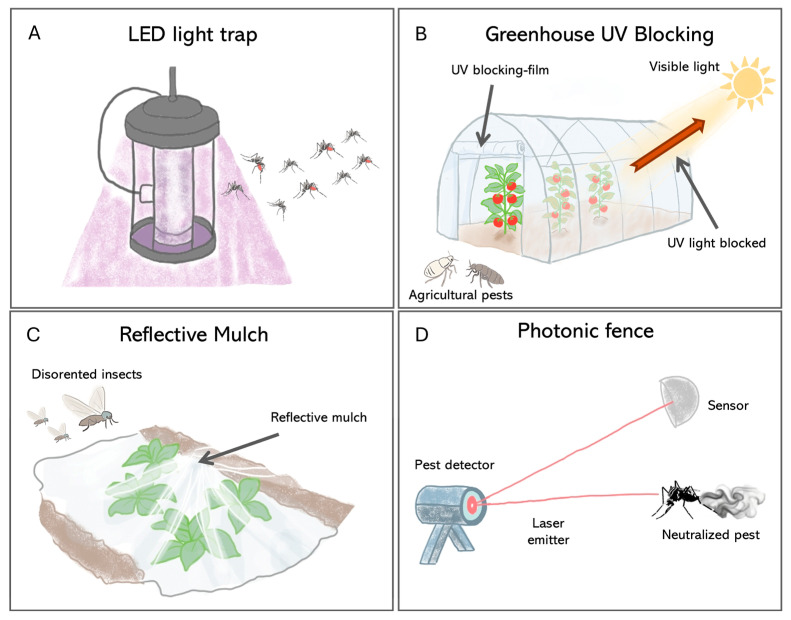
Pest control strategies based on the manipulation of light. (**A**) This method uses LED lights tuned to specific wavelengths—drawing in bugs that move toward light and then trapping them. (**B**) In greenhouses, special films block UV rays but let visible light pass—this disrupts how pests like *Bemisia tabaci* (Hemiptera, Aleyrodidae) locate hosts without harming plant growth. (**C**) Reflective mulch modifies the light environment around crops; upwardly reflected sunlight confuses airborne insects so they avoid settling on crops. (**D**) The photonic fence represents an active control system that uses sensors to detect insects and a laser pulse to neutralize it in real-time.

This is best illustrated using light traps, which have evolved from broad-spectrum UV and incandescent devices to energy-efficient LED systems. Because insects perceive colors differently from humans, especially responding to UV, blue, or green ranges [110], traditional incandescent bulbs are suboptimal. Modern LEDs, however, can be tailored to emit specific wavelengths that match the known visual sensitivities of target insects like mosquitoes and phlebotomine sand flies, a targeted approach that has been shown to increase capture rates by up to 50% (Figure 4A) [111,112,113].

More recently, advanced LED technology has further refined this approach by showing that while unmodulated UV light drastically enhances trapping for pests such as the Coconut Rhinoceros Beetle, *Oryctes rhinoceros* (Coleoptera, Scarabaeidae), and the Asian Citrus Psyllid, *Diaphorina citri* (Hemiptera, Psyllidae), specific visible wavelengths can have divergent effects—attracting psyllids via blue, yellow, and amber colors while suppressing beetle captures, thus enabling highly species-specific control strategies [114].

Beyond utilizing light as an attractant, light can also be used to suppress behavior. For instance, nocturnal pests can be inhibited by yellow or green light, which disrupts essential behaviors such as feeding and oviposition, while other wavelengths of light can act as repellents, effectively deterring insects from entering protected areas.

Indeed, beyond active light sources, passive optical interventions that modify the ambient visual environment are also effective. For example, UV-blocking plastic films can make greenhouses less visible to pests like whiteflies and thrips by disrupting their orientation and host-finding behaviors (Figure 4B), thus reducing infestation [115,116,117]. Similarly, reflective mulches interfere with the dorsal light response that many flying insects depend on for flight stabilization, thereby decreasing crop colonization rates (Figure 4C).

A different use applies computer-based visual systems to bypass human sensory limits that once restricted the development of logical tools [118,119]. By modeling the specific photoreceptor responses of a target insect, it is possible to engineer optimized visual lures. This approach has been used to design superior-colored traps for tse-tse fly (*Glossina fuscipes*; Diptera, Glossinidae) and Western flower thrip (*Frankliniella occidentalis*; Thysanoptera, Thripidae) control [120,121]. The future of this approach lies in the further development of such models to incorporate other key aspects of insect vision, such as their perception of polarized light from trap surfaces and how their characteristically low spatial acuity affects the detection of trap shapes from a distance [118,122].

The frontier in this field is the development of intelligent optical systems that integrate real-time surveillance with lethal intervention. A key example is the “Photonic Fence” (Figure 4D). This robotic approach utilizes machine vision to recognize and track flying insect vectors and a retina-safe laser to neutralize them mid-flight [123,124]. Field trials have shown that this method can selectively eliminate target pests such as *A. aegypti* and *Diaphorina citri* (Hemiptera, Psyllidae) with more than 97% efficacy while leaving non-target insects unharmed [124]. Since its mode of action is thermal damage, it precludes resistance evolution common with chemical insecticides [125]. These kinds of technologies allow for the creation of a nonchemical zone for vector interception and provide high-resolution surveillance data, a new paradigm for the protection of public health and agriculture [126].

Taken together, these innovations give an excellent example of progress, from broad light-based methods to precise, vision-guided technologies of targeted, greener pest control practices.

While our understanding of insect vision has grown enormously, from the kinetics of phototransduction to the circuits for motion detection, conveying such knowledge into effective pest control remains a formidable challenge. Although significant progress has been made in computational modeling and LED technology, a substantial amount of related research continues to employ overly simplified spectral sensitivity profiles. Current modeling approaches frequently overlook critical intra-specific variations, such as sexual dimorphism in *Aedes* spp. [127], as well as the synergistic interactions between visual, olfactory, and polarization cues. Moreover, there are few long-term field efficacy studies. Research primarily focuses on model organisms, such as *Drosophila* and *Apis* [128], while overlooking medically important vectors. Finally, trap design often overlooks the physiological limits of the insect compound eye. Its poor spatial resolution (1–2° vs. 0.01° in humans) makes it hard for insects to resolve shapes and distances [129], a fact that current predictive models ignore.

The use of visual technology in the control of pests presents its own set of challenges, including the development of resistance to the laser technology used in the Photonic Fence, scalability costs for subsistence farming in developing nations, and the impact on non-target pollinators [124]. However, the prospects are promising. The synergy between “vision-based” genetic modification for sterile insect carriers and LED drones could help lower chemical insecticide consumption by 50–70% by 2030. Alongside Machine Intelligence for live surveillance, this “visual–sterile insect technique” offers a sustainable model for dengue and malaria control in the face of the increasing threat in the EU.

To overcome these limitations in future studies, there is an imperative need to pursue key critical priorities: (1) the development of multi-modal fusion objects (visuo-olfactory) by AI to create “smart” traps that can respond to environmental stimuli [129,130]; (2) conducting genomic and CRISPR studies on larval opsins to identify specific targets [131], such as RNAi silencing of *Aaop3* in mosquitoes; (3) Confirmation of results in various species by connectomics, mapping key disease vectors; and (4) developing eco-specific 3D models accounting for intricate details such as crepuscular phototaxis and retinal micromovements to predict behavior accurately [129].

## 7. Conclusions

The insect compound eye is a striking example of evolutionary adaptation and morphological diversity, shaped by the unique ecological and behavioral demands of each species. Its role in mediating vital behaviors such as navigation, mate finding, and predator-prey interactions attests to its evolutionary importance. The major architectural types we described (apposition, optical superposition, and neural superposition eyes) represent distinct optical strategies for handling environmental challenges, such as obtaining high diurnal acuity or increasing nocturnal sensitivity and motion detection. This functional sophistication is not only about structural diversity across species but also about neural organization within a single insect. In fact, insects use visual information in a modular way, assigning specialized regions of the visual system and brain circuits to specific tasks.

Historically, efforts to utilize insect vision in practical applications, such as pest management, have been limited by human-centric biases, especially in designing traps and lures based on human color preferences. More recently, advances in computational modeling have overcome these limitations by simulating visual stimuli from the insect’s viewpoint, guided by species-specific photoreceptor sensitivities. This approach has led to the rational design of highly effective visual traps for pests such as *Glossina* spp. [132] and *F. occidentalis* [133], confirming that insect visual preferences diverge considerably from human intuition [120].

Beyond applied entomology, the study of insect vision greatly improves our understanding of the core principles that control neural circuit design. It is not surprising that many key organizational features—such as the parallel processing pathways for different visual types, hierarchical synaptic refinement across brain regions, and the modular tiling of the visual field—are conserved across various phyla in a way similar to vertebrate visual systems. These striking parallels suggest that evolutionary pressures have independently converged upon the most optimal strategies for processing visual information. Thus, the insect eye is more than just a model for ecological adaptation; it is a robust system for uncovering general principles in neurobiology. Easy to access, amenable to experimental manipulation, and evolutionarily diverse, it provides a unique tool for understanding the rules that govern the development and function of complex neural circuits—lessons that are ultimately crucial to also understanding the architecture of the human brain. Nevertheless, several significant challenges and opportunities remain. Future models of vision will need to incorporate additional aspects of insect visual perception, such as sensitivity to polarized light and the effects of low spatial resolution on shape detection at ecologically relevant distances. These factors must be incorporated into the next generation of species-specific, vision-based control technologies.

## Figures and Tables

**Table 1:** Overview of adaptive visual diversity across major insect orders.

Taxon	Visual System & Key Adaptation	Ecological Context	References
Odonata (*Anax junius*, *Sympetrum striolatum*)	Apposition. Huge eyes (~30k ommatidia); dorsal foveas with flattened facets for acute upward vision	Diurnal Predation. Aerial interception; tracking fast prey against bright sky	[42,43]
Orthoptera (*Schistocerca gregaria*, *Gryllus campestris*)	Apposition (Modified in Nocturnal). DRA for whole-sky polarization; neural summation for night sensitivity	Navigation & Migration. Celestial polarization compass; long-distance orientation	[44,45]
Hemiptera (*Notonecta glauca*, *Bemisia tabaci*)	Apposition. Aquatic zones for split underwater/aerial vision; polarization sensitivity to water surfaces	Amphibious/Agricultural. Water body detection via polarization; host-plant color recognition	[46,47]
Mantodea (*Tenodera sinensis*, *Mantis religiosa*)	Apposition. Frontal binocular overlap; “pseudopupil” fovea for high-acuity fixation	Ambush Predation. Stereopsis (depth perception) for precise close-range striking	[48,49]
Blattodea (*Periplaneta americana*, *Reticulitermes flavipes*)	Superposition (roaches) or reduced (termites). Wide rhabdoms for photon capture; often vestigial in termites	Scavenging/Subterranean. Navigation in extreme low light; reliance on non-visual senses	[50,51]
Coleoptera (*Scarabaeus zambesianus*, *Tribolium castaneum*)	Variable. Split eyes (whirligigs); moonlight polarization sensors; spectral tuning to bioluminescence	Niche-Specific. Nocturnal orientation via faint cues; intraspecific light signaling	[11,40]
Strepsiptera (*Stylops melittae*, *Xenos vesparum*)	Raspberry Eye. Clusters of large eyelets forming independent images; extreme sexual dimorphism (females blind)	Parasitic Life. Males rely on high-contrast vision solely to locate cryptic females quickly	[52,53]
Hymenoptera (*Apis mellifera*, *Cataglyphis fortis*)	Apposition. Trichromacy (UV-Blue-Green); DRA skylight compass; enlarged facets in nocturnal bees	Foraging & Homing. Landmark navigation; path integration; flower identification	[54,55]
Lepidoptera (*Papilio xuthus*, *Manduca sexta*)	Apposition (Day)/Superposition (Night). High spectral diversity (15+ receptors); tapetum lucidum in moths	Pollination & Signaling. Host-plant finding; mating cues (wing patterns); dim-light flight	[56,57]
Diptera (*Drosophila melanogaster*, *Anopheles gambiae*)	Neural Superposition. Open rhabdom (high sensitivity + acuity); specialized motion-tracking neurons (LPTCs)	Fast Flight & Vectoring. Aerobatics; landing control; visual-olfactory host seeking	[58,59]

## Data Availability

No new data were created or analyzed in this study. Data sharing is not applicable to this article.

## Abbreviations

The following abbreviations are used in this manuscript:

GRN	Gene Regulatory Network
PLC	Phospholipase C
PIP_2_	Phosphatidylinositol 4,5-bisphosphate
DAG	Diacylglycerol
IP_3_	Inositol 1,4,5-trisphosphate
TRP	Transient Receptor Potential (ion channel)
TRPL	TRP-like (ion channel)
PRC	Photoreceptor cell
DRA	Dorsal rim area
LGMD	Lobula Giant Movement Detector
DCMD	Descending Contralateral Movement Detector
LC4	Lobula Columnar (neuron)
LPLC2	Lobula–Plate Lobula Columnar (neuron)
GF	Giant Fiber (escape pathway)
LPTC	Lobula Plate Tangential Cell

## References

[1] Tomsic D., Theobald J. (2017). Insect Neurobiology: An Eye to Forward Motion. Curr. Biol..

[2] Fischer S., Muller C.H.G., Meyer-Rochow V.B. (2011). How Small Can Small Be: The Compound Eye of the Parasitoid Wasp *Trichogramma evanescens* (Westwood, 1833) (Hymenoptera, Hexapoda), an Insect of 0.3- to 0.4-Mm Total Body Size. Vis. Neurosci..

[3] Polilov A.A. (2017). Anatomy of Adult *Megaphragma* (Hymenoptera: *Trichogrammatidae*), One of the Smallest Insects, and New Insight into Insect Miniaturization. PLoS ONE.

[4] Warrant E., Kelber A., Kristensen N.P. (2003). Eyes and Vision. Band 4: Arthropoda, 2 Hälfte: Insecta, Lepidoptera, Moths and Butterflies, Teilband/Part 36, Vol 2: Morphology, Physiology, and Development.

[5] Olberg R.M., Seaman R.C., Coats M.I., Henry A.F. (2007). Eye Movements and Target Fixation during Dragonfly Prey-Interception Flights. J. Comp. Physiol. A.

[6] Kittelmann M., McGregor A.P. (2024). Looking across the Gap: Understanding the Evolution of Eyes and Vision among Insects. BioEssays.

[7] Buschbeck E.K., Friedrich M. (2008). Evolution of Insect Eyes: Tales of Ancient Heritage, Deconstruction, Reconstruction, Remodeling, and Recycling. Evol. Educ. Outreach.

[8] Giglio A., Vommaro M.L., Agostino R.G., Lo L.K., Donato S. (2022). Exploring Compound Eyes in Adults of Four Coleopteran Species Using Synchrotron X-Ray Phase-Contrast Microtomography (SR-PhC Micro-CT). Life.

[9] Paukner D., Wildenberg G.A., Badalamente G.S., Littlewood P.B., Kronforst M.R., Palmer S.E., Kasthuri N. (2024). Synchrotron-source Micro-x-ray Computed Tomography for Examining Butterfly Eyes. Ecol. Evol..

[10] Paulus H.F. (1972). Die Feinstruktur Der Stirnaugen Einiger Collembolen (Insecta, Entognatha) und Ihre Bedeutung für Die Stammesgeschichte Der Insekten. J. Zool. Syst. Evol. Res..

[11] Friedrich M. (2006). Ancient Mechanisms of Visual Sense Organ Development Based on Comparison of the Gene Networks Controlling Larval Eye, Ocellus, and Compound Eye Specification in *Drosophila*. Arthropod Struct. Dev..

[12] van Kleef J., Berry R., Stange G. (2008). Directional Selectivity in the Simple Eye of an Insect. J. Neurosci..

[13] Fuller S.B., Karpelson M., Censi A., Ma K.Y., Wood R.J. (2014). Controlling Free Flight of a Robotic Fly Using an Onboard Vision Sensor Inspired by Insect Ocelli. J. R. Soc. Interface.

[14] Buschbeck E.K. (2014). Escaping Compound Eye Ancestry: The Evolution of Single-Chamber Eyes in Holometabolous Larvae. J. Exp. Biol..

[15] Beutel R.G., Goczał J., Pohl H. (2025). Evolutionary Adaptations in Larvae of Holometabola. Annu. Rev. Entomol..

[16] Liu Z., Friedrich M. (2004). The Tribolium Homologue of Glass and the Evolution of Insect Larval Eyes. Dev. Biol..

[17] Fischer S., Laue M., Müller C.H.G., Meinertzhagen I.A., Pohl H. (2021). Ultrastructural 3D Reconstruction of the Smallest Known Insect Photoreceptors: The Stemmata of a First Instar Larva of Strepsiptera (Hexapoda). Arthropod Struct. Dev..

[18] Gilbert C. (1994). Form and Function of Stemmata in Larvae of Holometabolous Insects. Annu. Rev. Entomol..

[19] Meyer-Rochow V.B. (1974). Structure and Function of the Larval Eye of the Sawfly, Perga. J. Insect Physiol..

[20] Toh Y., Mizutani A. (1994). Neural Organization of the Lamina Neuropil of the Larva of the Tiger Beetle (*Cicindela chinensis*). Cell Tissue Res..

[21] Ichikawa T., Tateda H. (1982). Distribution of Color Receptors in the Larval Eyes of Four Species of Lepidoptera. J. Comp. Physiol. A.

[22] Stowasser A., Rapaport A., Layne J.E., Morgan R.C., Buschbeck E.K. (2010). Biological Bifocal Lenses with Image Separation. Curr. Biol..

[23] Rocha M., Kimler K.J., Leming M.T., Hu X., Whaley M.A., O’Tousa J.E. (2015). Expression and Light-Triggered Movement of Rhodopsins in the Larval Visual System of Mosquitoes. J. Exp. Biol..

[24] Nilsson D.-E., Kelber A. (2007). A Functional Analysis of Compound Eye Evolution. Arthropod Struct. Dev..

[25] Strausfeld N.J., Ma X., Edgecombe G.D., Fortey R.A., Land M.F., Liu Y., Cong P., Hou X. (2016). Arthropod Eyes: The Early Cambrian Fossil Record and Divergent Evolution of Visual Systems. Arthropod Struct. Dev..

[26] Paterson J.R., Edgecombe G.D., García-Bellido D.C. (2020). Disparate Compound Eyes of Cambrian Radiodonts Reveal Their Developmental Growth Mode and Diverse Visual Ecology. Sci. Adv..

[27] Clarkson E.N.K., Levi-Setti R. (1975). Trilobite Eyes and the Optics of Des Cartes and Huygens. Nature.

[28] French A.S. (1980). The Linear Dynamic Properties of Phototransduction in the Fly Compound Eye. J. Physiol..

[29] Harzsch S., Hafner G. (2006). Evolution of Eye Development in Arthropods: Phylogenetic Aspects. Arthropod Struct. Dev..

[30] Ready D.F., Hanson T.E., Benzer S. (1976). Development of the *Drosophila* Retina, a Neurocrystalline Lattice. Dev. Biol..

[31] Wolff T., Ready D.F. (1991). The Beginning of Pattern Formation in the *Drosophila* Compound Eye: The Morphogenetic Furrow and the Second Mitotic Wave. Development.

[32] Friedrich M., Rambold I., Melzer R.R. (1996). The Early Stages of Ommatidial Development in the Flour Beetle *Tribolium castaneum* (Coleoptera; Tenebrionidae). Dev. Genes Evol..

[33] Hafner G.S., Tokarski T.R. (1998). Morphogenesis and Pattern Formation in the Retina of the Crayfish *Procambarus clarkii*. Cell Tissue Res..

[34] Müller C.H.G., Rosenberg J., Richter S., Meyer-Rochow V.B. (2003). The Compound Eye of *Scutigera coleoptrata* (Linnaeus, 1758) (Chilopoda: Notostigmophora): An Ultrastructural Reinvestigation That Adds Support to the Mandibulata Concept. Zoomorphology.

[35] Misof B., Liu S., Meusemann K., Peters R.S., Donath A., Mayer C., Frandsen P.B., Ware J., Flouri T., Beutel R.G. (2014). Phylogenomics Resolves the Timing and Pattern of Insect Evolution. Science.

[36] Wernet M.F., Perry M.W., Desplan C. (2015). The Evolutionary Diversity of Insect Retinal Mosaics: Common Design Principles and Emerging Molecular Logic. Trends Genet..

[37] Agi E., Langen M., Altschuler S.J., Wu L.F., Zimmermann T., Hiesinger P.R. (2014). The Evolution and Development of Neural Superposition. J. Neurogenet..

[38] Feuda R., Marlétaz F., Bentley M.A., Holland P.W.H. (2016). Conservation, Duplication, and Divergence of Five Opsin Genes in Insect Evolution. Genome Biol. Evol..

[39] Backhaus W. (1992). The Bezold-Brücke Effect in the Color Vision System of the Honeybee. Vis. Res..

[40] Jackowska M., Bao R., Liu Z., McDonald E.C., Cook T.A., Friedrich M. (2007). Genomic and Gene Regulatory Signatures of Cryptozoic Adaptation: Loss of Blue Sensitive Photoreceptors through Expansion of Long Wavelength-Opsin Expression in the Red Flour Beetle *Tribolium castaneum*. Front. Zool..

[41] Srinivasan M.V. (2011). Honeybees as a Model for the Study of Visually Guided Flight, Navigation, and Biologically Inspired Robotics. Physiol. Rev..

[42] Land M.F., Nilsson D.-E. (2012). Animal Eyes.

[43] Sherk T.E. (1978). Development of the Compound Eyes of Dragonflies (Odonata). III. Adult Compound Eyes. J. Exper-Imental Zool..

[44] Homberg U. (2015). Sky Compass Orientation in Desert Locusts—Evidence from Field and Laboratory Studies. Front. Behav. Neurosci..

[45] Labhart T. (1996). How Polarization-Sensitive Interneurones of Crickets Perform at Low Degrees of Polarization. J. Exp. Biol..

[46] Schwind R. (1991). Polarization Vision in Water Insects and Insects Living on a Moist Substrate. J. Comp. Physiol. A.

[47] Warrant E., Nilsson D.-E. (2006). Invertebrate Vision.

[48] Rossel S. (1983). Binocular Stereopsis in an Insect. Nature.

[49] Prete F.R. (1999). The Praying Mantids.

[50] Meyers R.A. (2004). Encyclopedia of Molecular Cell Biology and Molecular Medicine.

[51] Weckstrom M., Jarvilehto M., Heimonen K. (1993). Spike-like Potentials in the Axons of Nonspiking Photoreceptors. J. Neurophysiol..

[52] Buschbeck E.K., Ehmer B., Hoy R.R. (2003). The Unusual Visual System of the Strepsiptera: External Eye and Neuropils. J. Comp. Physiol. A.

[53] Maksimovic S., Layne J.E., Buschbeck E.K. (2007). Behavioral Evidence for Within-Eyelet Resolution in Twisted-Winged Insects (Strepsiptera). J. Exp. Biol..

[54] Backhaus W. (1991). Color Opponent Coding in the Visual System of the Honeybee. Vision Res..

[55] Wakakuwa M., Kurasawa M., Giurfa M., Arikawa K. (2005). Spectral Heterogeneity of Honeybee Ommatidia. Naturwissenschaften.

[56] Warrant E. (2004). Vision in the Dimmest Habitats on Earth. J. Comp. Physiol. A.

[57] Arikawa K., Mizuno S., Kinoshita M., Stavenga D.G. (2003). Coexpression of Two Visual Pigments in a Photoreceptor Causes an Abnormally Broad Spectral Sensitivity in the Eye of the Butterfly Papilio Xuthus. J. Neurosci..

[58] Hardie R.C. (1986). The Photoreceptor Array of the Dipteran Retina. Trends Neurosci..

[59] Bate M., Martinez Arias A. (1993). The Development of Drosophila Melanogaster.

[60] Warren J., Kumar J.P. (2023). Patterning of the *Drosophila* Retina by the Morphogenetic Furrow. Front. Cell Dev. Biol..

[61] Salvemini M., Mauro U., Velaeti S., Polito C., Saccone G. (2006). A New Minos Vector for Eye-specific Expression of White+ Marker in *Ceratitis capitata* and in Distantly Related Dipteran Species. Insect Mol. Biol..

[62] Truman J.W., Riddiford L.M. (2019). The Evolution of Insect Metamorphosis: A Developmental and Endocrine View. Philos. Trans. R. Soc. B Biol. Sci..

[63] Melzer R.R., Paulus H.F. (2009). Evolutionswege Zum Larvalauge Der Insekten -Die Stemmata Der Höheren Dipteren Und Ihre Abwandlung Zum Bolwig-Organ. J. Zool. Syst. Evol. Res..

[64] Hu W., Wan D., Yu X., Cao J., Guo P., Li H., Han J. (2012). Protein Gq Modulates Termination of Phototransduction and Prevents Retinal Degeneration. J. Biol. Chem..

[65] Macias-Muñoz A., Murad R., Mortazavi A. (2019). Molecular Evolution and Expression of Opsin Genes in *Hydra vulgaris*. BMC Genom..

[66] Hardie R.C., Juusola M. (2015). Phototransduction in *Drosophila*. Curr. Opin. Neurobiol..

[67] Kiselev A., Subramaniam S. (1994). Activation and Regeneration of Rhodopsin in the Insect Visual Cycle. Science.

[68] Stavenga D.G. (1995). Insect Retinal Pigments: Spectral Characteristics and Physiological Functions. Prog. Retin. Eye Res..

[69] Hardie R.C., Postma M. (2008). Phototransduction in Microvillar Photoreceptors of *Drosophila* and Other Invertebrates. The Senses: A Comprehensive Reference.

[70] Katz B. (2009). *Drosophila* Photoreceptors and Signaling Mechanisms. Front. Cell. Neurosci..

[71] Yau K.-W., Hardie R.C. (2009). Phototransduction Motifs and Variations. Cell.

[72] Paulk A.C., Phillips-Portillo J., Dacks A.M., Fellous J.-M., Gronenberg W. (2008). The Processing of Color, Motion, and Stimulus Timing Are Anatomically Segregated in the Bumblebee Brain. J. Neurosci..

[73] Koyanagi M., Shen B., Nagata T., Sun L., Wada S., Kamimura S., Kage-Nakadai E., Terakita A. (2022). High-Performance Optical Control of GPCR Signaling by Bistable Animal Opsins MosOpn3 and LamPP in a Molecular Property–Dependent Manner. Proc. Natl. Acad. Sci. USA.

[74] Martin G.J., Lower S.E., Suvorov A., Bybee S.M. (2021). Molecular Evolution of Phototransduction Pathway Genes in Nocturnal and Diurnal Fireflies (Coleoptera: Lampyridae). Insects.

[75] Rind F.C. (2024). Recent Advances in Insect Vision in a 3D World: Looming Stimuli and Escape Behaviour. Curr. Opin. Insect Sci..

[76] Warrant E.J. (2017). The Remarkable Visual Capacities of Nocturnal Insects: Vision at the Limits with Small Eyes and Tiny Brains. Philos. Trans. R. Soc. B Biol. Sci..

[77] Kelber A., Balkenius A., Warrant E.J. (2002). Scotopic Colour Vision in Nocturnal Hawkmoths. Nature.

[78] Warrant E., Dacke M. (2011). Vision and Visual Navigation in Nocturnal Insects. Annu. Rev. Entomol..

[79] Honkanen A., Immonen E.-V., Salmela I., Heimonen K., Weckström M. (2017). Insect Photoreceptor Adaptations to Night Vision. Philos. Trans. R. Soc. B Biol. Sci..

[80] Schnaitmann C., Pagni M., Meyer P.B., Steinhoff L., Oberhauser V., Reiff D.F. (2024). Horizontal-Cell like Dm9 Neurons in *Drosophila* Modulate Photoreceptor Output to Supply Multiple Functions in Early Visual Processing. Front. Mol. Neurosci..

[81] Chen P.-J., Belušič G., Arikawa K. (2020). Chromatic Information Processing in the First Optic Ganglion of the Butterfly *Papilio xuthus*. J. Comp. Physiol. A.

[82] van der Kooi C.J., Stavenga D.G., Arikawa K., Belušič G., Kelber A. (2021). Evolution of Insect Color Vision: From Spectral Sensitivity to Visual Ecology. Annu. Rev. Entomol..

[83] Hassenstein B., Reichardt W. (1956). Systemtheoretische Analyse Der Zeit-, Reihenfolgen- Und Vorzeichenauswertung Bei Der Bewegungsperzeption des Rüsselkäfers Chlorophanus. Z. Naturforschung B.

[84] Borst A. (2014). Neural Circuits for Motion Vision in the Fly. Cold Spring Harb. Symp. Quant. Biol..

[85] Borst A., Drews M., Meier M. (2020). The Neural Network behind the Eyes of a Fly. Curr. Opin. Physiol..

[86] Nicholas S., Leibbrandt R., Nordström K. (2020). Visual Motion Sensitivity in Descending Neurons in the Hoverfly. J. Comp. Physiol. A.

[87] Rosner R., Tarawneh G., Lukyanova V., Read J.C.A. (2020). Binocular Responsiveness of Projection Neurons of the Praying Mantis Optic Lobe in the Frontal Visual Field. J. Comp. Physiol. A.

[88] Fenk L.M., Avritzer S.C., Weisman J.L., Nair A., Randt L.D., Mohren T.L., Siwanowicz I., Maimon G. (2022). Muscles That Move the Retina Augment Compound Eye Vision in *Drosophila*. Nature.

[89] Cheng K.Y., Frye M.A. (2020). Neuromodulation of Insect Motion Vision. J. Comp. Physiol. A.

[90] Gür B., Sporar K., Lopez-Behling A., Silies M. (2020). Distinct Expression of Potassium Channels Regulates Visual Response Properties of Lamina Neurons in *Drosophila melanogaster*. J. Comp. Physiol. A.

[91] Wang H., Foquet B., Dewell R.B., Song H., Dierick H.A., Gabbiani F. (2020). Molecular Characterization and Distribution of the Voltage-Gated Sodium Channel, Para, in the Brain of the Grasshopper and Vinegar Fly. J. Comp. Physiol. A.

[92] Freas C.A., Plowes N.J.R., Spetch M.L. (2019). Not Just Going with the Flow: Foraging Ants Attend to Polarised Light Even While on the Pheromone Trail. J. Comp. Physiol. A.

[93] Dacke M., Nordström P., Scholtz C.H. (2003). Twilight Orientation to Polarised Light in the Crepuscular Dung Beetle *Scarabaeus zambesianus*. J. Exp. Biol..

[94] el Jundi B., Smolka J., Baird E., Byrne M.J., Dacke M. (2014). Diurnal Dung Beetles Use the Intensity Gradient and the Polarization Pattern of the Sky for Orientation. J. Exp. Biol..

[95] Dacke M., Baird E., Byrne M., Scholtz C.H., Warrant E.J. (2013). Dung Beetles Use the Milky Way for Orientation. Curr. Biol..

[96] Freas C.A., Spetch M.L. (2023). Varieties of Visual Navigation in Insects. Anim. Cogn..

[97] Zeil J., Narendra A., Stürzl W. (2014). Looking and Homing: How Displaced Ants Decide Where to Go. Philos. Trans. R. Soc. B Biol. Sci..

[98] Zeil J., Fleischmann P.N. (2019). The Learning Walks of Ants (Hymenoptera: Formicidae). Myrmecol. News.

[99] Egelhaaf M. (2023). Optic Flow Based Spatial Vision in Insects. J. Comp. Physiol. A.

[100] Tammero L.F., Dickinson M.H. (2002). Collision-Avoidance and Landing Responses Are Mediated by Separate Pathways in the Fruit Fly, *Drosophila melanogaster*. J. Exp. Biol..

[101] Kern R., Boeddeker N., Dittmar L., Egelhaaf M. (2012). Blowfly Flight Characteristics Are Shaped by Environmental Features and Controlled by Optic Flow Information. J. Exp. Biol..

[102] Srinivasan M.V., Zhang S., Altwein M., Tautz J. (2000). Honeybee Navigation: Nature and Calibration of the “Odometer”. Science.

[103] Van Hateren J.H., Schilstra C. (1999). Blowfly Flight and Optic Flow: II. Head Movements during Flight. J. Exp. Biol..

[104] Supple J.A., Pinto-Benito D., Khoo C., Wardill T.J., Fabian S.T., Liu M., Pusdekar S., Galeano D., Pan J., Jiang S. (2020). Binocular Encoding in the Damselfly Pre-Motor Target Tracking System. Curr. Biol..

[105] Lecoeur J., Dacke M., Floreano D., Baird E. (2019). The Role of Optic Flow Pooling in Insect Flight Control in Cluttered Environments. Sci. Rep..

[106] Matsushita A., Stewart F., Ilić M., Chen P.-J., Wakita D., Miyazaki N., Murata K., Kinoshita M., Belušič G., Arikawa K. (2022). Connectome of the Lamina Reveals the Circuit for Early Color Processing in the Visual Pathway of a Butterfly. Curr. Biol..

[107] Chua N.J., Makarova A.A., Gunn P., Villani S., Cohen B., Thasin M., Wu J., Shefter D., Pang S., Xu C.S. (2023). A Complete Reconstruction of the Early Visual System of an Adult Insect. Curr. Biol..

[108] Rossi M., Hausmann A.E., Alcami P., Moest M., Roussou R., Van Belleghem S.M., Wright D.S., Kuo C.-Y., Lozano-Urrego D., Maulana A. (2024). Adaptive Introgression of a Visual Preference Gene. Science.

[109] Rana R., Kumawat M., Vishvendra, Kumawat R., Kumar R. (2024). Advancements in Insect Phototaxis and Its Implications for Pest Management: A Comprehensive Review. Uttar Pradesh J. Zool..

[110] Briscoe A.D., Chittka L. (2001). The Evolution of Color Vision in Insects. Annu. Rev. Entomol..

[111] Wilton D.P., Fay R.W. (1972). Air Flow Direction and Velocity in Light Trap Design. Entomol. Exp. Appl..

[112] Douglas A., Burkett J.F.B. (2005). Laboratory Evaluation of Colored Light as an Attractant for Female *Aedes aegypti*, *Aedes albopictus*, *Anopheles quadrimaculatus*, and *Culex nigripalpus*. Fla. Entomol..

[113] Mellor H.E., Hamilton J.G.C. (2003). Navigation of *Lutzomyia longipalpis* (Diptera: Psychodidae) under Dusk or Starlight Conditions. Bull. Entomol. Res..

[114] Paryavi M., Weiser K., Melzer M., Crook D., Ramadugu C., Jenkins D.M. (2025). Programmable LED Array for Evaluating Artificial Light Sources to Improve Insect Trapping. Insects.

[115] Costa H.S., Robb K.L. (1999). Effects of Ultraviolet-Absorbing Greenhouse Plastic Films on Flight Behavior of *Bemisia argentifolii* (Homoptera: Aleyrodidae) and *Frankliniella occidentalis* (Thysanoptera: Thripidae). J. Econ. Entomol..

[116] Antignus Y., Nestel D., Cohen S., Lapidot M. (2001). Ultraviolet-Deficient Greenhouse Environment Affects Whitefly Attraction and Flight-Behavior. Environ. Entomol..

[117] Kumar P., Poehling H.-M. (2006). Uv-Blocking Plastic Films and Nets Influence Vectors and Virus Transmission on Greenhouse Tomatoes in the Humid Tropics. Environ. Entomol..

[118] Caves E.M., Brandley N.C., Johnsen S. (2018). Visual Acuity and the Evolution of Signals. Trends Ecol. Evol..

[119] Santer R.D., Allen W.L. (2024). Optimising the Colour of Traps Requires an Insect’s Eye View. Pest Manag. Sci..

[120] Santer R.D., Okal M.N., Esterhuizen J., Torr S.J. (2021). Evaluation of Improved Coloured Targets to Control Riverine Tsetse in East Africa: A Bayesian Approach. PLoS Negl. Trop. Dis..

[121] Dearden A.E., Wood M.J., Frend H.O., Butt T.M., Allen W.L. (2024). Visual Modelling Can Optimise the Appearance and Capture Efficiency of Sticky Traps Used to Manage Insect Pests. J. Pest Sci..

[122] Meglič A., Ilić M., Pirih P., Škorjanc A., Wehling M.F., Kreft M., Belušič G. (2019). Horsefly Object-Directed Polarotaxis Is Mediated by a Stochastically Distributed Ommatidial Subtype in the Ventral Retina. Proc. Natl. Acad. Sci. USA.

[123] Keller M.D., Norton B.J., Farrar D.J., Rutschman P., Marvit M., Makagon A. (2020). Optical Tracking and Laser-Induced Mortality of Insects during Flight. Sci. Rep..

[124] Patt J.M., Makagon A., Norton B., Marvit M., Rutschman P., Neligeorge M., Salesin J. (2024). An Optical System to Detect, Surveil, and Kill Flying Insect Vectors of Human and Crop Pathogens. Sci. Rep..

[125] Liu N. (2015). Insecticide Resistance in Mosquitoes: Impact, Mechanisms, and Research Directions. Annu. Rev. Entomol..

[126] Benelli G., Beier J.C. (2017). Current Vector Control Challenges in the Fight against Malaria. Acta Trop..

[127] Blake A.J., Riffell J.A. (2025). Spectral Preferences of Mosquitos Are Altered by Odors. J. Exp. Biol..

[128] Preti M., Verheggen F., Angeli S. (2021). Insect Pest Monitoring with Camera-Equipped Traps: Strengths and Limitations. J. Pest Sci..

[129] Roberts N.S., Jones M., Shah F., Butt T.M., Allen W.L. (2025). Modeling Spatial Acuity Improves Trap Capture of Western Flower Thrips, *Frankliniella occidentalis* (Thysanoptera: Thripidae). J. Insect Sci..

[130] Santer R.D., Allen W.L. (2025). Insect Visual Perception and Pest Control: Opportunities and Challenges. Curr. Opin. Insect Sci..

[131] Kumar H., Gal’chinsky N., Sweta V., Negi N., Filatov R., Chandel A., Ali J., Oberemok V., Laikova K. (2025). Perspectives of RNAi, CUADb and CRISPR/Cas as Innovative Antisense Technologies for Insect Pest Control: From Discovery to Practice. Insects.

[132] Santer R.D., Vale G.A., Tsikire D., Torr S.J. (2019). Optimising Targets for Tsetse Control: Taking a Fly’s-Eye-View to Improve the Colour of Synthetic Fabrics. PLoS Negl. Trop. Dis..

[133] van Tol R.W.H.M., Davidson M.M., Butler R.C., Teulon D.A.J., de Kogel W.J. (2020). Visually and Olfactorily Enhanced Attractive Devices for Thrips Management. Entomol. Exp. Appl..

